# The Impact of Stigma, Autism Label and Wording on the Perceived Desirability of the Online Dating Profiles of Men on the Autism Spectrum

**DOI:** 10.1007/s10803-020-04830-8

**Published:** 2021-01-18

**Authors:** M. Brosnan, J. Gavin

**Affiliations:** Department of Psychology, University of Bath, Claverton Down, Bath, BA2 7AY UK

**Keywords:** Autism, Stigma, Online dating, Attractiveness

## Abstract

Those seeking romantic relations are increasingly using online dating sites, including young men on the autism spectrum. This study presented dating profiles with and without an explicit label of autism and positive or negative wording to 306 ‘females seeking a male partner’. Participants assessed the men’s dating profiles in terms of perceived attractiveness, trustworthiness and desire-to-date. They also completed a questionnaire on their level of stigmatisation of, and familiarity with, autism. An explicit autism label and positive wording positively impacted perceived attractiveness. With positively worded profiles, those with highly stigmatising views reported decreased desire-to-date when an explicit label of autism was present; those with low levels of stigmatising reported increased desire-to-date when an explicit autism label was present.

Despite strong desires to form romantic relationships, adults on the autism spectrum can experience a range of challenges in successfully initiating romance (Hancock et al. 2019; Sala et al. 2020). One specific challenge is an apparent formation of less favourable first impressions of adults on the autism spectrum by the neurotypical population (Cage and Burton 2019; Morrison et al. 2019; Sasson et al. 2017; Sasson and Morrison 2019). One area where this may be particularly challenging is online dating, in which first impressions are based on an online dating profile. Indeed, one of the reported dilemmas faced by online daters on the autism spectrum is whether or not to include their diagnostic label in their profile (Roth and Gillis 2015). This is a common question faced by many intellectually able adults on the autism spectrum generally, who believe that camouflaging autism may enable fitting in and avoiding discrimination and negative responses from others (Cage and Troxell-Whitman 2019; Hull et al. 2017). This study explored the role of stigmatisation towards autism on the perceived desirability of online dating profiles that either did or did not contain an explicit label of autism.

Sasson et al. (2017) conducted a number of studies examining first impressions of adults on the autism spectrum compared to first impressions of a matched sample of neurotypical adults. Sasson et al. found that adults on the autism spectrum were rated as more awkward, less attractive, less likeable, and less desirable for social interaction than their neurotypical counterparts. However, ratings were more favourable when an autism label was provided and when observers had greater knowledge about autism, suggesting that diagnostic disclosure, in some scenarios, may help rather than harm social evaluation of adults on the autism spectrum. This may be because a diagnostic label provides raters with an explanation for atypical first impressions (Morrison et al. 2019). Thus, adults on the autism spectrum who miss out on social opportunities because of negative judgements by their neurotypical peers may benefit from disclosing their diagnosis (Morrison et al. 2019; Sasson et al. 2017). This is supported by research showing that the first impressions of neurotypical people are also rated as more favourable when they were labelled as having an autism diagnosis (for experimental purposes, they did not have a diagnosis in reality; Morrison et al. 2019). This is consistent with other research which has highlighted that the presence of diagnostic information can reduce negative perceptions of autism behaviours (Brosnan and Mills 2016; Butler and Gillis 2011; Matthews et al. 2015). Sasson et al.s’ patterns of findings concerning first impressions are remarkably robust, occur within seconds, do not change with increased exposure, persist across both child and adult age groups, and across neurotypical raters and raters on the autism spectrum (DeBrabander et al. 2019; Sasson et al. 2017). Importantly, Sasson and Morrison (2019) also found that, whilst an explicit autism label improved evaluation of first impressions of adults on the autism spectrum, they remained significantly lower than the evaluations of neurotypical comparisons.

An additional factor to consider when disclosing diagnosis is stigma. Stigma is elicited by negative attitudes towards characteristics that deviate from accepted cultural norms, and recognition of such characteristics is the first step in the enactment of stigma (Goffman 1963). Link and Phelan (2001, 2006), following Goffman (1963), conceptualized stigma in terms of a four-stage process: Firstly people identify and label differences they observe in others; secondly, people make assumptions (stereotypes) about the labelled group and extend those assumptions to all the individuals in the group; thirdly people distance themselves from the labelled group; and finally people use these stereotypes to discriminate against individuals in the labelled group (see Kinnear et al. 2016 for an account of the impact of stigmatisation towards autism). Morrison et al. (2019) report that the positive effects of disclosing an autism diagnosis did not extend to neurotypical people who held stigmatising views towards autism. Indeed, for neurotypical raters with high stigmatisation towards autism, the addition of an explicit autism label *worsened* evaluations of the first impressions. Stigma associated with autism can be elicited by two different sets of discriminative features: The explicit label of autism itself and behaviours associated with autism (Butler and Gillis 2011; Someki et al. 2018; see Mitter et al. 2019 for systematic review). Stigmatising views towards autism have largely been assessed at the third stage of Link and Phelan’s model through the social distancing scale, which asks how willing people would be to engage in a series of behaviours with someone on the autism spectrum (move next door to, spend an evening socialising with, collaborate with, befriend, have marry into family, marry/date oneself; Gillespie-Lynch et al. 2015). Although two of the items on the social distancing scale refer to marriage/dating, how stigma relates to potential romantic relationships is unknown. Gillespie-Lynch et al. (2017) shed some light on this by comparing scores on the social distancing scale between respondents on the autism spectrum and neurotypical respondents. Whilst both groups expressed relatively little stigma to the first four items (move next door to, spend an evening socialising with, collaborate with, befriend) the neurotypical group demonstrated significantly greater stigma towards the final two items relating to romance (have marry into family, marry/date oneself). Similarly, in a large scale survey of stigmatisation towards autism amongst US and Japanese college students, Someki et al. (2018) found that while the overall level of stigmatisation towards autism was relatively low, more than half of the participants reported an unwillingness to date or marry someone on the autism spectrum. This suggests that stigmatisation towards autism is particularly marked when the social interaction involves romantic relationships.

In terms of seeking romantic relationships, many adults on the autism spectrum report turning to online dating, with one study reporting that 53% of adults on the autism spectrum have engaged in online dating compared to 15% of the general population (Roth and Gillis 2015). Traditional online dating platforms^1^ present potential romantic partners with a ‘dating profile’ to inform first impressions. Typically, online dating profiles provide a profile picture, a brief demographic profile and a description of interests and activities, upon which first impressions are based. Gavin et al. (2019a) analysed a wide range of dating profiles posted by males on the autism spectrum who were seeking a female romantic partner.^2^ The authors report that whilst the norm of a dating profile is to present an optimum version of oneself and what one is seeking in a potential partner (Toma and Hancock 2010; Whitty 2008), the dating profiles posted by males on the autism spectrum violated this norm and were frequently negative in tone. Males on the autism spectrum were likely to describe their negative attributes, underplay their strengths, and highlight what they did not want from a potential partner (Gavin et al. 2019a). In a subsequent study, Gavin et al. (2019b) developed positively and negatively worded variants of the same behaviours and interests that had been frequently posted on online dating sites by males on the autism spectrum, and also varied whether an explicit label of autism was present or not. The authors found that both positive wording and an explicit statement of an autism diagnosis enhanced perceived attractiveness and trustworthiness, but not desirability-to-date. Participants were ‘females seeking a male’, each rating one dating profile, and even though each profile had the same profile picture, the presence of an autism label related to increased perceived physical attractiveness specifically. The authors speculated that this effect may relate to the association between physical attractiveness and trustworthiness, and that disclosing a diagnosis of autism enhances trustworthiness. Similarly, whilst those on the autism spectrum were generally perceived negatively in Sasson and Morrison’s (2019) study, this did not extend to trustworthiness, which did not differ from neurotypical comparisons.

It is clear that the way male online daters on the autism spectrum present themselves and their autism (or not) impacts on aspects of females’ perception of attractiveness, trustworthiness and desirability-to-date. Moreover, the way that males on the autism spectrum present themselves in online dating profiles is a clear deviation from online dating norms (Gavin et al. 2019a; Toma and Hancock 2010; Whitty 2008), and thus may lead to stigmatising views towards online daters on the autism spectrum. As observed by Goffman (1963), stigma is elicited by negative attitudes towards characteristics that deviate from accepted cultural norms, and recognition of difference is the first step in the enactment of stigma (Link and Phelan 2001, 2006). Therefore the present study sought to identify the impact of relatively high or low social distancing scores (as an index of stigmatising views) on females’ perceptions of the attractiveness (physical, social, task), trustworthiness and desire-to-date of male online dating profiles. There were seven hypotheses. Hypothesis 1: There will be a main effect for autism label, specifically, the presence of an autism label will have a positive impact on evaluations. Hypothesis 2: There will be a main effect for wording, specifically, positive wording will have a positive effect on evaluations. Hypothesis 3: There will be a main effect of stigma, specifically, higher levels of stigmatising will have a negative effect on evaluations. In addition, the independent variables were hypothesised to interact with each other. Hypothesis 4: Autism label and stigma will interact with each other, specifically the provision of an explicit label of autism will have a negative impact on the evaluations from those with highly stigmatising views towards autism. Hypothesis 5: Wording and stigma will interact with each other, specifically, positive wording to describe autism behaviours/ interests will have a positive impact on the evaluations from those with low stigmatising views towards autism. Hypothesis 6: Autism label and wording will interact with each other, specifically, the provision of an explicit label and positive wording will have a positive impact on evaluations. Finally, there will be a three way interaction—Hypothesis 7: Autism Label and negative wording will have a negative impact on evaluations of those with highly stigmatising views.

## Method

### Participants

Invitations to participate in a study of online dating profiles were circulated via undergraduate social media platforms (such as university bulletin boards). Inclusion criteria were being a female seeking a male, aged 18–25, and residing in the UK. The survey was completed by 366 participants. As 28 were not residing in the UK, they were excluded from the analysis, leaving 334 participants. After the manipulation, a catch question was included to confirm that participants were aware of whether autism had been explicitly mentioned in the profile (see below). This was identified correctly by 306 participants whose responses were analysed (83.6% of original sample). Chi^2^ analysis confirmed that the excluded participants did not differ from the included participants with respect to the conditions they were assigned to (described below; label: Chi^2^(1) = 0.22, ns; wording chi^2^(1) = 1.85, ns). T-test analysis confirmed that the excluded participants did not differ from the included participants on any of the experimental variables, except they did perceive less trustworthiness (3.7 vs 3.9, t(364) = 2.06, p < .05). Included participants had a mean age of 19.53 years (SD = 1.11, range: 18–24). 81.0% were ‘White’, 13.7% ‘Asian’, 2.6% ‘Mixed ethnic groups’, 0.7% ‘Black’ and 2% from ‘other ethnic groups’.

### Design

Within the UK context, the identity-first terminology of ‘autistic’ is preferred by members of the autistic community (Kenny et al. 2016) and was used in the measures. The design replicated the previous research of Gavin et al. (2019b), with the addition of the social distancing scale (Gillespie-Lynch et al. 2015). To be included in the study, participants were asked to confirm their sex as female and that they would seek a male if they were in a dating context. Participants also reported their age, ethnicity and which country they were currently residing in.

Participants were randomly presented with one of four dating profiles following a 2 (autism label: present/absent) by 2 (wording: positive/negative) design. Each profile was identical, with the same profile photograph and biographical information, body type, and height). The male image was created by combining two young adult white European male images posed under standardised lighting and with a neutral expression. The composite image was made by averaging and then combining the shape and colour information of the two individual facial photographs using specialised software. The image was rated as 3.5 for attractiveness, the midpoint on a 0–7 scale (this technique follows previous studies, see Gavin et al. 2019b).

In addition to this, embedded in behaviours/interests were five phrases that were either positively worded (I am happy in my own company; when I do something I like to give it my undivided attention; I like my conversations to have a point to them; I have good attention to detail; I am a practical down-to-earth thinker) or negatively worded (I do not find social situations easy; I'm not very good at multi-tasking; I don't like social chit-chat; I can tend to miss ‘The Bigger Picture’; I am not very imaginative). These were taken from analysis of the dating profiles of men on the autism spectrum and have been used in previous research (Gavin et al. 2019a, b). Two profiles (one positively worded, one negatively worded) had the addition of an explicit statement ‘I am autistic’ at the end of their profile.

Participants then completed the following rating scales all of which had Likert scale response options (strongly disagree to strongly agree) with mean scores that could range from 1 to 5.

Social attractiveness (McCroskey et al. 2006). Social attractiveness had 12 items such as “He would be sociable with me”. Six of these items were reversed scored. Chronbach's alpha = .93 suggesting the scale, social attractiveness, has excellent internal consistency. A higher score indicates higher perceived social attractiveness.

Physical attractiveness (McGloin and Denes 2018). Physical attractiveness was measured through perceptions of the profile image with three items, such as “I find this person physically attractive”. The scale has good internal consistency Chronbach's alpha = .87. A higher score indicates higher perceived physical attractiveness.

Task attractiveness (McCroskey and McCain 1974). Task attraction was measured via five items, such as “He would be a typical loser when assigned a job”. Chronbach's alpha = .83 indicating good internal consistency. A higher score indicates higher perceived task attractiveness.

Trustworthiness (McCroskey and Teven 1999). This scale contained six items, some of which were reversed scored, such as “I think he would be untrustworthy”. Chronbach's alpha = .87 indicating good internal consistency. A higher score indicates higher perceived trustworthiness.

Desire-to-date (McGloin and Denes 2018). Desire-to-date measured whether the respondent thought the individual was desirable and would want something to develop after viewing the profile. There were five items such as “This person would be a very desirable date”. Chronbach's alpha = .96 indicating excellent internal consistency. A higher score indicates higher perceived desire to date.

After reading the profiles, participants completed ratings of social, task and physical attractiveness, trustworthiness and desire-to-date. Participants were then asked if the profile had explicitly stated that they were autistic (response options: yes, no, don’t know). It was then explained that the researchers had an interest in raising awareness of autism, stating ‘Autism is characterised by challenges with social communication and interaction combined with repetitive behaviours, activities or interests. Up to 2% of the population are thought to be autistic.’ Participants were then asked to complete the ‘experience of autism scale’ (Gardiner and Iarocci 2014). Scores were the highest level of experience, and could range from 1 (I have never observed a person that I was aware was autistic) to 12 (I am autistic).

Finally, participants completed the six-item social distancing scale as an index of stigmatising (Gillespie-Lynch et al. 2015). The four response options ranged from ‘definitely willing’ through to ‘definitively unwilling’, so scores could range from 6 to 24. The reliability of this scale is good with Cronbach’s alpha = 0.86 (Morrison et al. 2019). A higher score indicates higher levels of stigmatising views. For the analysis, the total stigma score was split around the mean into low (6–9) and high (10–22). The mean split method was used to explore the interactions with label and wording, enabling comparison with previous research (Gavin et al. 2019b). Whilst this meant that some low stigma scores (9) were similar to high stigma scores (10), this was only the case for 11% of each group, and the means between the two groups were significantly different (t(304) = 24.50, p < .001).

### Analysis

Means were calculated for all the five dependent variables. If a data point was missing, the mean was calculated from the remaining data points (unless more than 50% were missing, but this did not occur. In fact, there were very few missing data points). A MANCOVA analysis was planned with attractiveness (social, task, physical) trustworthiness and desire-to-date as the five dependent variables. The independent variables were autism label (present/absent), wording (positive/negative) and stigmatising views (high/low). 53% had an autism label and 47% had no autism label; 53% had positive wording profiles and 47% had negative wording profiles; 52% had low stigma, 48% had high stigma. Experience was a covariate. The assumptions for the MANCOVA were checked and met for outliers, multivariate normality and multicollinearity. The homogeneity of covariance assumption was not met. Levene’s Test identified greater variance in task attractiveness in the ‘negative wording’ condition compared to the ‘positive wording’ condition and greater variance in trustworthiness in the ‘positive wording’ condition compared to the ‘negative wording’ condition (this assumption was met for all other comparisons). As the assumption was met for 8 of the 10 comparisons and greater variance was not consistently in one condition, this violation was noted (see 'Discussion') and the MANCOVA analysis undertaken. The analyses were run using SPSS (version 26). Ethical approval for the study was obtained through the Psychology Research Ethics Committee at the University of Bath.

## Results

The means, standard deviations and ranges of the ratings for the five dependent variables for the whole group are in Table 1. The highest level of experience had a median and mode of Level 9 (21.2%: A friend of the family is autistic) followed by Level 10 (19.3%: I have a relative who is autistic), Level 6 (13.4%: I have worked with an autistic person at my place of employment (or study)), Level 3 (11.8%: I have watched a movie or television show in which a character depicted is an autistic person) and Level 11 (10.5%: I live with an autistic person). The means and standard deviations for the four (label × wording) conditions are in Table 2. Whilst there were no differences in number, age or stigma (all p > .05) across the 4 conditions, the difference in experience did reach significance (F(1,303) = 4.01, p < .05).

**Table 1:** Descriptive statistics (n = 306): means, standard deviations (SD) and ranges

Physical_attractiveness mean	2.97 (SD = 1.0; range: 1.00–5.00)
Social_attractiveness mean	3.39 (SD = 0.70; range: 1.33–4.92)
Task_attractiveness mean	3.69 (SD = 0.67; range 1.40–5.00)
Trustworthiness_mean	3.89 (SD = .56; range: 2.17–5.00)
Desire-to-date_mean	2.32 (SD = .99; range: 1.00–4.80)
Stigma_total	9.73 (SD = 3.00; range: 6–22)
Highest_experience	7.40 (SD = 2.86; range 1–12)

**Table 2:** Means (and standard deviations) by condition

	Label positive wording	Label negative wording	No label positive wording	No label negative wording
Number	74	89	70	73
Age	19.54 (1.15)	19.53 (1.14)	19.58 (1.13)	19.48 (1.04)
Experience	6.84 (2.90)	7.31 (2.94)	7.69 (2.91)	7.82 (2.64)
Stigma	9.77 (2.95)	9.61 (2.86)	9.83 (3.30)	9.74 (2.97)

Wilks' Lambda indicated that the MANCOVA model was significant for all three independent variables (Label: F(5, 292) = 4.77, p < .001, η_p_^2^ = .075; Wording: F(5,292) = 18.79, p < .001, η_p_^2^ = .243; Stigma: F(5,292) = 3.20, p = .008, η_p_^2^ = .052). The presence of an explicit autism label was associated with increased perceived physical attractiveness (F(1, 296) = 3.71, p = .055, η_p_^2^ = .012), social attractiveness (F(1,296) = 4.70, p = .03, η_p_^2^ = .016), task attractiveness (F(1,296) = 12.83, p < .001, η_p_^2^ = .042), and trustworthiness (F(1,296) = 8.44, p = .004, η_p_^2^ = .028) of the profile (compared to an absence of label) but not desire-to-date (F(1,296) = 0.03, p = .89, η_p_^2^ = .000). Hypothesis 1 was therefore accepted for four of the dependent variables. Positive wording was also associated with increased perceived social attractiveness (F(1,296) = 48.14, p < .001, η_p_^2^ = .140), task attractiveness (F(1,296) = 44.27, p < .001, η_p_^2^ = .130) and desire-to-date (F(1,296) = 30.48, p < .001, η_p_^2^ = .093) of the profile (compared to negative wording) but not perceived physical attractiveness (F(1,296) = 2.23, p = .14, η_p_^2^ = .007) nor trustworthiness (F(1,296) = 0.43, p = .51, ηp^2^ = .001). Hypothesis 2 was therefore accepted for three of the dependent variables. Highly stigmatising views were associated with decreased perceived social attractiveness (F(1,296) = 7.22, p = .008, η_p_^2^ = .024) and task attractiveness (F(1,296) = 7.03, p = .008, η_p_^2^ = .023) of the profile (compared to low levels of stigmatising), but not perceived physical attractiveness (F(1,296) = .12, p = .73, η_p_^2^ = .000), trustworthiness (F(1,296) = 0.01, p = .92, η_p_^2^ = .000) nor desire-to-date (F(1,296) = 1.05, p = .31, η_p_^2^ = .004). Hypothesis 3 was therefore accepted for two of the dependent variables.

**Fig. 1:**
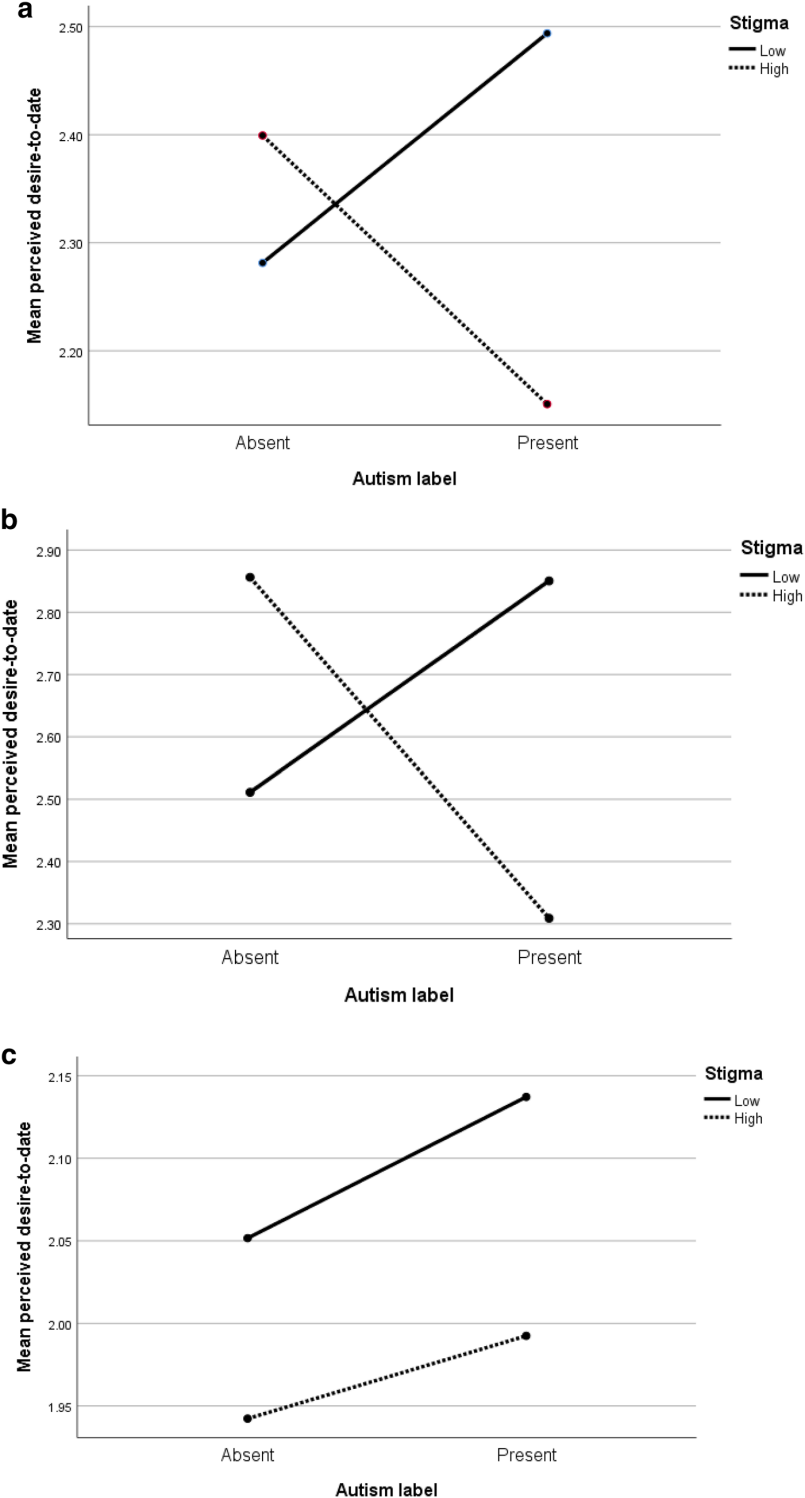
**a** 2-way interaction between label and stigma on perceived desire-to-date. **b** 3-way interaction between label and stigma on perceived desire-to-date for positive wording. **c** 3-way interaction between label and stigma on perceived desire-to-date for negative wording

Exploring the 2-way interactions, two were significant. Consistent with Hypothesis 4, stigmatising views significantly interacted with label; the presence of an autism label being associated with decreased perceived social attractiveness (F(1,296) = 8.04, p = .005, η_p_^2^ = .026) and decreased desire-to-date (F(1,296) = 4.49, p = .035, η_p_^2^ = .015) in those with high levels of stigmatising. Wording did not significantly interact with label or stigmatising views (Hypotheses 5 and 6). In line with Hypothesis 7, the 3-way interaction between stigmatising views, label and wording approached significance for desire-to-date (F(1,296) = 3.81, p = .052, η_p_^2^ = .013). Figure 1a highlights that the 2-way interaction for perceived desire-to-date arises from the presence of an autism label being associated with both an increase in perceived desire-to-date for those with low levels of stigmatising and a comparable decrease in desire-to-date for those with highly stigmatising views. Figure 1b highlights that this pattern is replicated in the three-way interaction for positive wording, but not for negative wording (Fig. 1c). Recall that there was a significant main effect for wording on desire-to-date and note the means in Figure in 1b are all higher than the means in Fig. 1c. This indicates that negative wording was associated with lower desire-to date, and label and stigmatising views had little impact. However, positive wording was associated with higher desire-to-date, and in this context the presence of an explicit autism label was associated with both increased perceived desire-to-date in those with low levels of stigmatising and decreased perceived desire-to-date in those with highly stigmatising views. Finally, the covariate of autism-experience was not significant (all p > .05), though autism-experience did significantly negatively correlate with the stigmatising views total (r = − .15, p = .007).

## Discussion

Males on the autism spectrum can desire romantic relationships and many use online dating as a forum for initiating potential romantic interaction. The online dating profile represents the initial impression of the dater, which formed the context of the present study. For the first time, this study identified the factors that impact upon the perceived desirability of males on the autism spectrum, with respect to raters’ levels of stigmatisation towards autism, the presence or absence of an explicit autism label, and positive or negative wording in the online dating profile. Overall, the presence of a specific autism label and positive wording was associated with a positive impact, and the presence of highly stigmatising views towards autism by the rater was associated with a negative impact on the perceived desirability of males on the autism spectrum. The three-way interaction approached significance, such that with positively worded dating profiles, the presence of an explicit autism label was associated with comparably decreased desirability for raters with highly stigmatising views and increased desirability for raters with low levels of stigmatising views.

The findings within this online dating context are comparable to those of Morrison et al. (2019). Both studies identified that those with highly stigmatising views evaluate individuals on the autism spectrum negatively with a reduced willingness for interaction. Importantly, the present study also found that low levels of stigmatisation did not equate to no effect, rather a relatively positive effect. This suggests that low levels of stigmatisation are not merely the absence of stigma, but the presence of relatively positive views about autism. Whether this represents a ‘positive stigma’ based upon comparable processes to (negative) stigma is an open question. Figure 1b (for positive wording) highlights that the ratings for the low stigmatisation group when a label was present were comparable to the high stigmatisation group when a label was absent. Whilst these ratings are relatively high, all the ratings have means around or below the mid-point of 3, indicating that on average, participants were neutral about desirability-to-date. Thus ‘positive stigma’ may be relative, in that negative consequences of stigma are eliminated rather than an absolute increase in attitudes towards people on the autism spectrum (see Sasson and Morrison 2019). With respect to the four-stage process of stigma (Link and Phelan 2001, 2006), positive stigma may share the same two first stages (identify and label differences; make assumptions (stereotypes) about the labelled group). Differences may emerge at the third stage—with a comparable/greater (as opposed to less) willingness to interact in future (and then potentially level 4 reflecting supportive rather than discriminatory behaviour). Positive stigma towards people on the autism spectrum may be based on positive stereotypes (e.g., people on the autism spectrum are inherently trustworthy, see also Sasson et al. 2017), which may in turn set up unrealistic expectations if a relationship develops.

Both an explicit label of autism and positive wording were associated with the dating profile being rated more positively for social and task attractiveness. Stigmatising views, on the other hand, were associated with negative perceptions of social and task attractiveness. The 2-way interactions highlighted that social attractiveness specifically was rated negatively by those with high stigmatisation when an explicit label of autism was present. High stigmatisation may impact upon social attractiveness specifically as the diagnostic criteria for autism focus upon social communication and interaction difficulties (APA 2013). This may reflect the use of the social distancing scale as our measure of stigmatisation which explicitly asks about willingness to interact. In addition, consistent with our prediction, an explicit label of autism was also associated with greater perceived trustworthiness. This may reflect the disclosure as a signal for honesty and trustworthiness, or may reflect a positive stereotype of (or positive stigma about) autism (Huntley et al. 2019; de Schipper et al. 2016). Similarly, characters on the autism spectrum in mainstream media are often portrayed with enhanced intellectual abilities (Draaisma 2009), which may engender a positive stereotype (or positive stigma) related to perceived task attractiveness in those with low stigmatisation (for example, see Stern and Barnes 2019).

The (main effect) findings are also consistent with Gavin et al. (2019b) who found an explicit label of autism was associated with greater perceived physical attractiveness (recall that the same photo is used for all profiles). This finding is consistent with Sasson and Morrison (2019) who found images of neurotypical faces were rated more desirable when they were given a (false) autism label and related research which has found an explicit autism label can reduce negative evaluations (Brosnan and Mills 2016; Butler and Gillis 2011; Matthews et al. 2015). Despite the positive evaluations of an explicit label of autism, this did not extend to desire-to-date, which is again consistent with previous research (Gavin et al. 2019b). The 3-way interaction highlighted that with positive wording, participants with low stigmatisation who were assigned to the autism label condition had the highest desire-to-date scores relative to other participants. The significance of positive wording is highlighted by the statistical model effect sizes, which showed that only wording had a large effect size (label and stigma had medium effect sizes). It should also be noted that the effect sizes for the interactions were small and that homogeneity of covariance assumptions were not met for wording (trustworthiness and task attractiveness only).

Such findings are contextualised within the literature which suggests that complying with implicit social norms relates to social success and that this extends from face-to-face interaction to online interaction. One of the benefits of online interaction for adults on the autism spectrum can be that time is afforded to reflect upon communication, enabling enough time to respond appropriately, rather than the rapid, spontaneous responses often required in face-to-face interactions (Brosnan and Gavin 2015; see Brosnan et al. 2016). Guidance for online daters on the autism spectrum could make explicit the implicit social norms of desirable online self-presentation, thereby providing the relevant information to make a positive first impression. The present research identifying a positive effect of an autism label on some evaluations is distinct from Sasson et al.’s (2017) finding that still/static images of individuals on the autism spectrum were evaluated less positively than images of neurotypical controls. Consistent with previous research, the image used in the present study was of an averaged face (see Gavin et al. 2019b). Future research can explore this distinction using a range of faces from those on the autism spectrum and neurotypical controls.

The study also has a range of limitations that need to be borne in mind. It has been suggested that younger, better educated, Western (compared to Middle or Far Eastern) females may be a group likely to be relatively lower in stigmatising views (Gillespie-Lynch et al. 2017; 2019; Someki et al. 2018; White et al. 2019). These findings are therefore likely limited to the UK culture of college-aged females. In addition, females have been found to value intelligence and ambition more while choosing a potential partner, whereas males place greater importance on physical appearance (see Jackson et al. 1995, for meta-analysis) suggesting that the pattern of findings may be limited to females seeking a male (see also Cage and Burton 2019). Much college-based autism research is likely to take place in universities that have an autism research focus, and it is possible that such a focus of research activity within these universities encourages more knowledge and acceptance of autism in students attending these universities (especially if based in the same department). Sasson and Morrison (2019) for example, note the very high autism knowledge scores of their student participants. The average level of experience with autism in the present sample was also relatively high. Consistent with other studies, whilst greater experience significantly correlated with reduced stigmatisation, the correlation was small. Future research can address such issues. Relatively high or low stigmatisation was operationalised by dividing the scores from the social distancing scale, rather than employing the measure as a continuous variable which has its limitations. Most participants had a friend of the family or relative or experience working with someone on the autism spectrum. However, the social distancing scale does not enquire about the characteristics of autism that the respondent may be drawing upon. Another limitation, therefore, is that there will likely be variability in the conceptions of autism between participants.

Assuming the aim of online dating is to initiate romance, the implications for males on the autism spectrum are clear: (1) use positive wording—which is consistent with the norms in the online dating context (Gavin et al. 2019a; Toma and Hancock 2010; Whitty 2008) and (2) be explicit about autism diagnoses (Gavin et al. 2019b). This is associated with positive evaluations from potential daters lower in stigmatising views. This may also reduce contact from people with highly stigmatising views, but anecdotal accounts indicate that highlighting an autism diagnosis does not result in fewer potential dates, rather more suitable dates (Sykes 2014). Importantly too, whilst an online dating profile might represent the quintessential first impression, future research can seek to support romantic engagement when it is desired (Hancock et al. 2019; Sala et al. 2020) beyond first impressions.

## Footnotes

1 Excluding dating apps such as Tinder.

2 On the basis that most diagnosed adults on the autism spectrum are heterosexual males.
